# Multi-scaled self-attention for drug–target interaction prediction based on multi-granularity representation

**DOI:** 10.1186/s12859-022-04857-x

**Published:** 2022-08-03

**Authors:** Yuni Zeng, Xiangru Chen, Dezhong Peng, Lijun Zhang, Haixiao Huang

**Affiliations:** 1 School of Information Science and Technology, Zhejiang Sci-Tech University, Hangzhou, China; 2 College of Computer Science, Sichuan University, Chengdu, China; 3 Shenzhen Peng Cheng Laboratory, Shenzhen, China; 4 Chengdu Sobey Digital Technology Co., Ltd, Chengdu, China; 5 Sichuan Zhiqian Technology Co., Ltd, Chengdu, China; 6 Chengdu Ruibei Yingte Information Technology Co., Ltd, Chengdu, China; 7 Sichuan Provincial Commission of Politics and Law, Chengdu, China

**Keywords:** Drug–target interaction, Deep learning, Self-attention networks, Representations learning

## Abstract

**Background:**

Drug–target interaction (DTI) prediction plays a crucial role in drug discovery. Although the advanced deep learning has shown promising results in predicting DTIs, it still needs improvements in two aspects: (1) encoding method, in which the existing encoding method, character encoding, overlooks chemical textual information of atoms with multiple characters and chemical functional groups; as well as (2) the architecture of deep model, which should focus on multiple chemical patterns in drug and target representations.

**Results:**

In this paper, we propose a multi-granularity multi-scaled self-attention (SAN) model by alleviating the above problems. Specifically, in process of encoding, we investigate a segmentation method for drug and protein sequences and then label the segmented groups as the multi-granularity representations. Moreover, in order to enhance the various local patterns in these multi-granularity representations, a multi-scaled SAN is built and exploited to generate deep representations of drugs and targets. Finally, our proposed model predicts DTIs based on the fusion of these deep representations. Our proposed model is evaluated on two benchmark datasets, KIBA and Davis. The experimental results reveal that our proposed model yields better prediction accuracy than strong baseline models.

**Conclusion:**

Our proposed multi-granularity encoding method and multi-scaled SAN model improve DTI prediction by encoding the chemical textual information of drugs and targets and extracting their various local patterns, respectively.

## Background

Drug–target interaction (DTI) indicates the binding of drug compounds to their targets. The targets refer to the proteins or some bio-molecules to which the drug directly binds, and which are responsible for the therapeutic efficacy of the drug in vivo [1]. The drugs exert their clinical effects in treating diseases by changing the structure of the targets or regulating their metabolism. Therefore, accurate identification of DTI is one crucial step of drug discovery and development [1–3]. For example, in process of drug repositioning [4] task, DTI prediction is regarded as the foundation to find new targets of existing drugs. Nowadays, due to the high-cost and time-consuming traditional biological experiments, effective computational methods are urgently needed [5–7].

In response to this demand, many DTI prediction methods have been proposed in recent years. These methods mainly includes two parts: encoding methods and DTI prediction methods.

As for the encoding methods, most studies for DTI prediction label their inputs by a character-based dictionary. For example, in DeepDTA [6], with a dictionary like {‘C’:1,‘H’:2,‘N’:3,$\ldots$,‘=’:63}, the drug simplified molecular input line entry system (SMILES) sequence ‘CN=C=O’ was labelled as [1 3 63 1 63 5]. It labelled each character of drug SMILES by its corresponding integer in the character-based dictionary. In addition, in other chemical compounds related fields, some works applied tokenization methods to extract substrings from drug sequences as their functional groups at the chemical level. Study [8] tokenized the names of chemical compounds by the open parser for systematic IUPAC nomenclature (OPSIN) tokenizer [9] and byte-pair-encoding (BPE) [10] in predicting chemical compounds task. Based on BPE, study [11] introduced a tokenization algorithm named SMILES pair encoding (SPE) to label the SMILES by the learned chemical groups. It has been applied to generative and predictive tasks and molecular tasks. Study [12] proposed a ChemBoost approach to predict protein-ligand binding affinity scores based on substrings extracted by Word2vec [13] and BPE. In these studies, tokenizer methods in the fields of natural language processing (NLP) were used for drug SMILES segmentation, and then the segmented SMILES were applied to compound-related tasks.

For DTI prediction methods,many efforts have been conducted to predict drug–target binding affinity scores in recent years. The traditional approach to DTI prediction mainly based on similarity [14, 15]. Study [16] used the 2D compound similarity of drugs and Smith-Waterman similarity of targets as the inputs. Then, the Kronecker regularized least squares (KronRLS) algorithm was employed to predict the binding affinity values of drug-tart pairs. Study [17] also utilized a number of similarity-based information and features to predict DTI by a gradient boosting machine. DTINet [18] was based on the assumption that similar drugs may share similar targets. Taking a series of similar matrices as input, it was designed to find an optimal projection from drug space onto target space by the random walk with restart (RWR) algorithm.

With the significant success of deep learning in computer version, speech recognition and NLP, deep learning models are widely used in DTI prediction. DeepDTA [6] employed two convolutional neural network (CNN) models to extract features for deep representations of drugs and targets. Then, an fully connected network was utilized to predict the interaction of drug and protein representations. OnionNet [19] also utilized CNNs for drug and protein representations and so as to predict the binding affinity values. GANsDTA [20] used the generative adversarial networks (GANs) to learn deep representations for drugs and targets, and then predicted the binding affinity scores of drug–target pairs. DeepCDA [21] also was proposed for binding affinity score prediction. It employed two CNNs to extract feature of drug and target. Then, long-short-term memory (LSTM) layers and a two-side attention mechanism were used in interaction learning to predict DTIs. Moreover, self-attention networks (SANs) also were applied to generate deep representations of drugs and targets [22–24]. Especially, study [23] proved that SANs have the ability to capture the long-distance relation between atoms in drug and target sequences.

Despite these efforts, the existing methods have several areas for improvement:

- The existing encoding method labels molecular input character by character and it cannot encode fundamental chemical groups: (1) atoms with multiple characters in compounds, like ‘Br’, ‘Cl’, and (2) chemical functional groups, like ‘CC’, ‘OH’. These chemical groups are the determining part of chemical compounds and protein sequences. Therefore, the existing encoding method leads to the loss of essential chemical information.
- The existing deep models do not fully model different chemical correlations between atoms and atoms, atoms and chemical groups, chemical groups and chemical groups. Although CNNs can capture local features of these correlations, they failed to model long-distant atoms [23]. Besides, SANS focus on the overall input sentence, but they may overlook fine-grained information in drug and target sequences [25]. Thus, the existing deep model for DTI prediction need to improve.

In order to address the above problems, we introduce a new multi-scaled SAN model for drug–target binding affinity prediction based on multi-granularity representations in this work. Taking protein sequences and drug SMILES sequences as inputs, we first introduce a multi-granularity encoding method for them. The multi-granularity encoding is built upon the BPE algorithm which is a widely used tokenization algorithm in field of NLP. BPE calculates the frequency of occurrence of each consecutive byte pair, and then forms a vocabulary from high-frequency byte pairs. The multi-granularity representations are labelled by the vocabulary and then transmitted as inputs to our proposed multi-scale SAN model. By assigning different window sizes to heads in SAN, the multi-scaled SAN is exploited to learn the multi-scaled local patterns and generate deep representations of drugs and targets. Finally, the prediction is made on fused deep representations.

To the end, we evaluate the effectiveness of our proposed model on benchmark datasets (Davis [26] and KIBA [27]). Experimental results demonstrate that our multi-granularity multi-scaled model yields better accuracy over baselines and existing DTI deep models. Moreover, the experiment analyses reveal that both the multi-granularity encoding and multi-scaled features extracted by our multi-scaled SANs are beneficial to DTI prediction.

## Methods

**Fig. 1:**
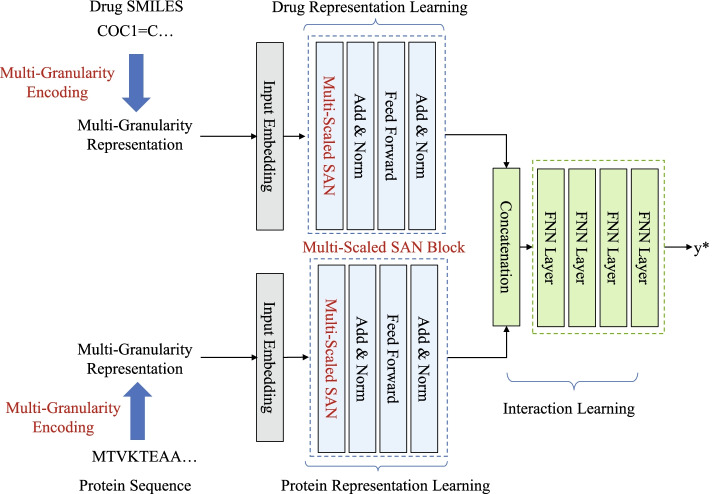
Our proposed multi-granularity multi-scaled SAN model for DTI prediction

In this work, we propose a multi-granularity multi-scaled method for DTI prediction, as shown in Fig. 1. The proposed method includes four components: multi-granularity encoding, drug representation learning, protein representation learning, and the interaction learning part. Firstly, we introduce a multi-granularity encoding method for drug and protein input sequences. In this process, the input sequences are encoded by a multi-granularity vocabulary, which are generated by a segmentation method. Then, taken the multi-granularity representations as inputs, a multi-scaled SAN is proposed to extract and fuse multi-scaled local features. Finally, the prediction is made on fused deep drug representations and deep protein representations by fully connected feed-forward networks.

### Multi-granularity encoding

The current labeling method is not sufficient to encode chemical sequences since it ignores the chemical textual information from chemical groups in drugs and proteins, for example, chemical functional groups ‘[C@@H]’,‘Br’. Thus, the intuitive way for representing a chemical sequence is to find out the substrings in the sequence by a computational method. Here, the substring is the chemical functional groups or atoms with multiple characters.

BPE [10] is a data compression method that can obtain high-frequency substrings to segment the sequence. In the field of NLP, BPE is widely used in different text tasks and as the first step to understand text sentences. BPE initializes the symbol vocabulary with the character vocabulary, and then it iteratively counts the frequency of adjacent character pairs in the corpus and merges the pair with the highest frequency to a new symbol. Finally, the vocabulary update is stopped when the number of merge operations reaches a threshold.

In this work, we utilize the BPE algorithm to generate vocabularies for encoding molecular inputs (SMILES or proteins). First, the segmentation datasets of drugs and targets are built and used to train BPE. Then, the BPE model trained by drug data would generate a vocabulary $V_d$ with a threshold $T_d$ for drugs, and $V_p$ and $T_p$ for targets. *T* determines the size of the generated vocabulary which consists of the segmented inputs by BPE. For example, taken the ‘COC1=C(C=C2C(= C1)N=CN=C2NC3=C(C(=CC=C3)Cl)F)CN4CCCC[C@@H]4C(=O)N’ as the input, the segmented outputs of BPE is shown in Table 1 with different *T*.

**Table 1:** Examples of segmented outputs of ’COC1=C(C=C2C(=C1)N=CN=C2NC3 =C(C(=CC=C3)Cl)F)CN4CCCC[C@@H]4C(=O)N’ with different *T*

*T*	Segmented SMILES Sequence (Vocabulary)
1*k*	COC1=C(C=C, 2C(=C1), N=C, N=C2, N, C3=C, (C(=CC=C,
	3)C, l), F)C, N, 4, CCCC, [C@@H]4, C(=O)N
5*k*	CO, C1=C, (C=C, 2, C(=C1), N=C, N=C, 2, N, C3=C, (,
	C(=CC=C, 3)C, l), F, )C, N, 4, CCCC, [C@@H], 4, C(=O), N
25*k*	C, O, C1=C, (C=C, 2, C(=C, 1), N=C, N=C, 2, N, C3,
	=C, (, C(, =CC=C, 3, )C, l, ), F, )C, N, 4, CCCC,
	[C@@H], 4, C(=O), N

Finally, a multi-granularity dictionary is constructed by assigning each group in the vocabulary a corresponding integer like the character-level dictionary in study [6]. Thus, an input sequence is labelled as multi-granularity representation $X =\{x_1,x_2,\ldots ,x_i,\ldots \}$ where $x_i \in N^*$ and the length of *X* is varied, which depends on the length of the input sequence.

### Multi-scaled self-attention model for drug–target binding affinity prediction

Our multi-scaled SAN is built upon Transformer block [28] which has shown excellent capability on sequence processing tasks. Given a drug multi-granularity representation $X_d$ and protein multi-granularity representation $X_p$, we first adopt an input embedding module to integrate multiple embeddings. Then, for drug embedding $E_d$ and protein embedding $E_p$, two multi-scaled SAN blocks are exploited to capture the local patterns features of drugs and proteins, respectively. Finally, an interaction block is proposed to fuse and extract interaction features from deep drug representations $R_d$ and deep protein representations $R_p$. The final prediction $y^*$ is the output of the interaction block.

#### Input embedding

Given a multi-granularity drug input as

1 $$\begin{aligned} X_d = \{d_1, d_2, \ldots , d_{l_d}\}, \end{aligned}$$

and a multi-granularity protein input as

2 $$\begin{aligned} X_p = \{p_1, p_2, \ldots , p_{l_p}\}, \end{aligned}$$

we define a hyper-parameter *l* to restrict the max input length. Specially, $l_d$ restricts drug input $X_d$ and $l_p$ restricts target input $X_p$. If the length of *X* is shorter than *l*, the lack value is setting as 0. According to Transformer [28] and MT-DTI [23], the input of multi-scaled SAN is the sum of token embedding $E_t$ of the input sequence and position embedding $E_p$ of the input sequence, that is calculated as:

3 $$\begin{aligned} E_d = E^d_t + E^d_p. \end{aligned}$$

Here, the token embedding $E^d_t \in {\mathbb{R}}^{l_d \times e_d}$ has a trainable weight $W^d_t\in {\mathbb{R}}^{v_d\times e_d}$. The $v_d$ is the vocabulary size of drugs and $e_d$ is the embedding length of drugs. The position embedding $E^d_p \in {\mathbb{R}}^{l_d \times e_d}$ has a trainable weight $W^d_p\in {\mathbb{R}}^{l_d \times e_d}$. As for protein embedding,

4 $$\begin{aligned} E_p = E^p_t + E^p_p. \end{aligned}$$

where $E^p_t \in {\mathbb{R}}^{l_p \times e_p}$ is the token embedding of $X_p$, $E^p_p \in {\mathbb{R}}^{l_p \times e_p}$ is the position embedding of $X_p$ and $e_p$ is the embedding size of protein sequence.

#### Multi-scaled self-attention block

**Fig. 2:**
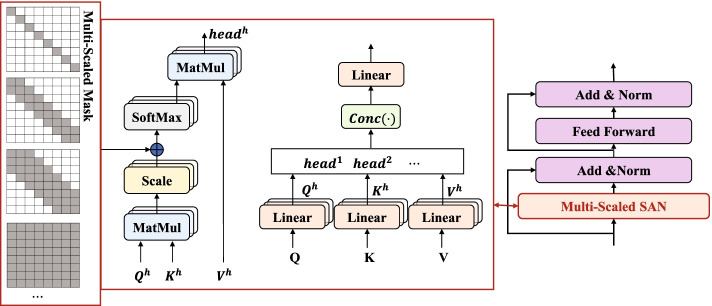
Our proposed multi-scaled SAN block

Multi-head SAN is the main component of Transformer [28]. It performs multiple self-attention modules on input expressions, then jointly pay attention to the information of different expression at different position. In this work, in order to generate a more informative deep representations of drugs and proteins, we adopt multi-scaled SAN to their embedings, which assign different window size to heads in multi-head SAN, that is formulated as,

5 $$\begin{aligned} R_d&= MSSAN(E_d,L_d), \end{aligned}$$

6 $$\begin{aligned} R_p&= MSSAN(E_p,L_p). \end{aligned}$$

where MSSAN($\cdot$) denotes a multi-scaled self-attention block, as shown in Fig. 2. $L_d$ and $L_p$ are the hyper-parameters notating the number of multi-scaled SAN blocks.

Especially, suppose the input to multi-scaled SAN blocks is *E*. Our model first transforms input sequence into *N* subspace with different linear projections,

7 $$\begin{aligned} Q^h,K^h,V^h = EW^h_Q, EW^h_K,EW^h_V, \end{aligned}$$

where $1\le h\in {{\mathbb{N}}}^+ \le N$ is the index and $W^h_* \in {{\mathbb{R}}}^{e_d \times d^h_*}$, the $d^h$ denotes the dimensionality of the $h^{th}$ head subspace. Then, we utilize a mask matrix $M^h \in {{\mathbb{R}}}^{l\times l}$ for the $h^{th}$ head to achieve multi-scaled SAN. The output of $h^{th}$ head on multi-scaled SAN is calculated as,

8 $$\begin{aligned} head^h = softmax \left( \frac{{Q^hK^h}^T}{\sqrt{d_h}} + M^h \right) V^h, \end{aligned}$$

where $M^h$ is determined by a hyper-parameter named window size $m^h$,

9 $$\begin{aligned} M^h_{i,j} = \left\{ \begin{aligned} -\infty ,&\ j <(i - m^h), \\ 0 ,&\ (i - m^h) \le j \le (i+m^h), \\ -\infty ,&\ j \>(i+m^h). \end{aligned} \right. \end{aligned}$$

Then, the *h* heads are concatenated,

10 $$\begin{aligned} heads = conc[head^1,\ldots ,head^h,\ldots ,head^N] \end{aligned}$$

where $conc(\cdot )$ is a concatenation function. Next, a residual connection [29] and the layer normalization (LN($\cdot$)) [30] are employed,

11 $$\begin{aligned} Z = LN(heads + E). \end{aligned}$$

Thus, the output of a multi-scaled SAN block is formulated,

12 $$\begin{aligned} MSSAN(E,1) = LN(Z + FFN(Z,1)), \end{aligned}$$

where *FFN*(*Z*, 1) denotes one fully connected feed-forward layer (FCN) with ReLU activation [31] and *Z* as input. The hidden size of the FCN is $e_d$.

### Interaction block

The interaction block in this work is to combine deep drug and protein representations and predicts the binding affinity scores of drug–target pairs. Mathematically, firstly,

13 $$\begin{aligned} R = conc[R_d,R_p]. \end{aligned}$$

Next, 4 layers of FCN are employed to capture the interaction information from *R*.

14 $$\begin{aligned} y* = FFN(R,4) \end{aligned}$$

where $y^*$ is the predicted binding affinity value of the drug–target pair.

## Data and experiments

### Datasets

#### Benchmark datasets for DIT prediction

We evaluated our proposed model on Davis [26] and KIBA [27] datasets because they are widely used in existing drug–target interaction studies. Specially, in order to ensure the uniqueness of drug input sequence, we only use Isomeric SMILES strings in this paper. The number of proteins, compounds and interactions of the Davis and KIBA dataset are summarised in Table 2. In particular, the Davis dataset contains the 442 kinase proteins, their relevant inhibitors (68 ligands) and their respective dissociation constant ($K_d$) value. The binding affinity scores of drug–target pairs were transformed $K_d$ into log space $pK_d$, as [6, 17], as follows,

15 $$\begin{aligned} pK_d = - log_{10}\left( \frac{{K_d}}{1e9}\right) . \end{aligned}$$

The used KIBA dataset comprised 229 proteins, 2111 drugs and their KIBA scores. Here, the KIBA scores measure the kinase inhibitor bioactivities as the binding affinity values in following experiments.

**Table 2:** The summary of the benchmark datasets

	Proteins	Compounds	Interactions	Training Data	Test Data
Davis	442	68	30056	25046	5010
KIBA	229	2111	118254	98545	19709

#### Segmentation dataset

We collect drug SMILES sequences from the National Center for Biotechnology Information (NCBI) 1 and protein sequences from The Universal Protein Resource^2^. Finally, 147546 SMILES sequences and 114500 protein sequences are collected as segmentation data to train the segmentation methods.

### Experiment setup and metric

Table 3 summaries other hyper-parameter settings. We use five-time leave-one-out cross-validation to train our model and list the average results on test data. All models were trained on 1 NVIDIA 3080 GPU.

**Table 3:** Summary of parameter settings

Parameter	KIBA	Davis
$l_d$	80	36
$l_p$	800	900
$m^h$	0,1,2,3	0,1,2,3
$e_d$	128	64
$L_d$	2	2
$L_p$	2	1
Hidden size in FFN	1024,1024,512,1	
Epoch	300	300
Dropout	0.1	0.1
Optimizer	Adam	Adam
Learning rate	0.0001	0.0001

To measure the performance of our model, three metrics are included: mean squared error (MSE), Concordance Index (CI) and the $r^2_m$ metric. MSE is the loss of the optimizer in the deep model.

16 $$\begin{aligned} MSE = \frac{{1}}{n} \sum _{i=1}^n (y^*_i - y_i)^2 \end{aligned}$$

where the $y^*$ is the predicted binding affinity value, *y* is the ground-truth and *n* is the number of drug–target pairs.

CI is the probability of the predicted scores of two randomly chosen drug–target pairs in the correct order, as

17 $$\begin{aligned} CI = \frac{{1}}{N} {\sum _{\delta _i > \delta _j}} f(t_i - t_j) \end{aligned}$$

where $t_i$ is the predicted value with larger affinity $\delta _i$, $t_j$ is the prediction score for smaller affinity $\delta _j$ and *N* is a normalization constant. Moreover, the *f*(*x*) is a step function [16],

18 $$\begin{aligned} f(x) = \left\{ \begin{aligned} 0&,&if \ x<0, \\ 0.5&,&if \ x=0, \\ 1&,&if \ x>0. \\ \end{aligned} \right. \end{aligned}$$

Then $r^2_m$ metric [32, 33] is another widely used metric in this filed. Mathematically,

19 $$\begin{aligned} r^2_m = r^2 * (1-\sqrt{r^2 - r^2_0}), \end{aligned}$$

where $r^2$ and $r^2_0$ are the squared correlation coefficient values between the observed and predicted values with and without intercept, respectively. Especially, the $r^2_m$ value of an acceptable model should be larger than 0.5.

### Experiments 1: Effects of the segmentation method

**Fig. 3:**
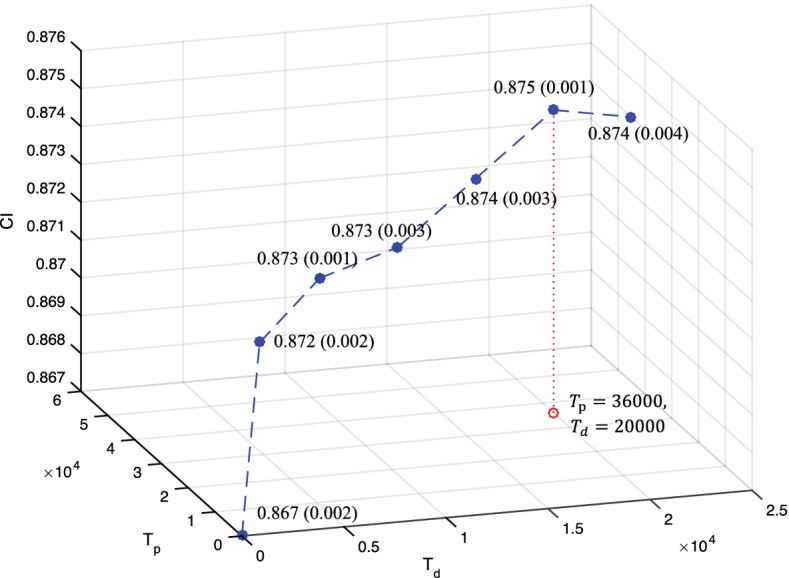
Results of DeepDTA [6] model on the KIBA dataset with different multi-granularity representations as inputs. These multi-granularity representations are encoded by BPE algorithm with different threshold *T*. Here, $T_d$ is the threshold *T* for drug segmentation and $T_p$ is the threshold *T* for protein segmentation

**Fig. 4:**
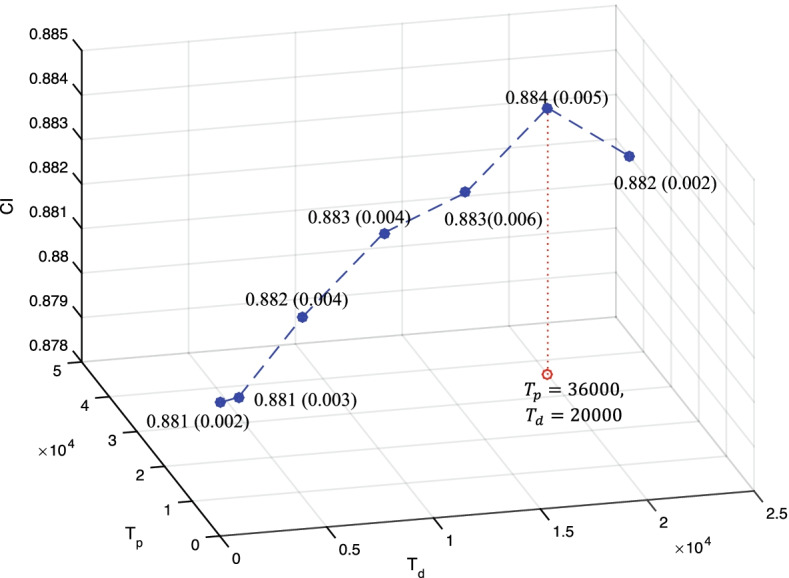
Results of DeepDTA [6] model on the Davis dataset with different multi-granularity representations as inputs. These multi-granularity representations are encoded by BPE algorithm with different threshold *T*. Here, $T_d$ is the threshold *T* for drug segmentation and $T_p$ is the threshold *T* for protein segmentation

In this paper, the BPE algorithm is utilized as the segmentation method to learn the substrings in drug SMILES and protein sequences. As seen in Table 1, the threshold *T* determines the degree of segmentation. The larger *T* in BPE indicates the more fine-grained and longer segmentation outputs. We first investigated the effects of *T* to DTI prediction on KIBA and Davis dataset. We extract various multi-granularity representations by setting different *T*, and then build DeepDTA [6] models with these representations as inputs. As plotted in Figs. 3 and 4, the prediction results on KIBA and Davis dataset are demonstrated, respectively.

*Discussion:* For both KIBA and Davis dataset, the $T_d = 20k$ and $T_g =36k$ is superior to other settings. It is clear that when $T_d < 20k$ and $T_g < 36k$, the prediction quality goes up as *T* increases. Conversely when $T_d > 20k$ and $T_g > 36k$, the increase of *T* seems to cause performance degradation. One possible reason is that the segmented SMILE with $T_d = 20k$ and the segmented protein sequences with $T_g =36k$ include more chemical textual information for predicting DTI. As the result, $T_d = 20k$ and $T_g =36k$ in following experiments.

### Experiments 2: Encoding methods for DTI prediction

The starting point of our approach is an observation in encoding methods. Considering the improvements of existing character-based encoding methods, we adopt segmentation method to learn the chemical groups in drug and target sequences. Thus, in this subsection, we evaluate whether deep representations learned from multi-granularity representations contains more drug–target interaction information than deep representations learned from character encoded representations, We also implemented DeepDTA [6], as baseline, with multi-granularity representations and character encoded representations as inputs. Table 4 lists the average results of the drug–target binding affinity prediction on KIBA and Davis dataset.

*Discussion:* As seen, the multi-granularity encoding method improves the prediction quality in both two datasets, reconfirming the necessity of encoding the chemical groups in drug and protein sequences.

**Table 4:** Results of DeepDTA (CNN model) on KIBA and Davis dataset with character-based and multi-granularity encoding. Especially, the character-based encoding methods is original labelling method in DeepDTA [6]

	Encoding Method	CI	MSE	$r^2_m$
KIBA	Character Encoding	0.863 (0.002)	0.194	0.673 (0.009)
	Multi-Granularity	**0.875 (0.001)**	**0.185**	**0.696 (0.017)**
Davis	Character Encoding	0.878 (0.004)	0.261	0.630 (0.017)
	Multi-Granularity	**0.884 (0.005)**	**0.250**	**0.655 (0.019)**

Bold values indicate the best results on the datasets

### Experiments 3: Multi-scaled SAN for DTI prediction

In this section, we conducted experiments about deep models based on multi-granularity encoding. Table 5 gives the average test results on the drug–target binding affinity prediction tasks. One intuition of our work is to capture the local patterns in multi-granularity representations by multi-scaled SANs. To evaluate it, we implemented models with CNNs from DeepDTA [6], SANs from Transformer [28] which also employed in MT-DTI [23] and our multi-scaled SAN.

*Discussion:* As shown in Table 5, the multi-scaled SAN outperforms the SANs model, indicating that the local pattern information can raise the ability of SANs to capture the drug–target interaction information. Moreover, as all known, CNNs have the ability to capture the local features. According to Table 5, the multi-scaled model achieved higher results than CNNs model, revealing extracting local features by the dynamic weights of multi-scaled SANs is superior to fixed weight from CNNs.

**Table 5:** Results of deep models on KIBA and Davis dataset with multi-granularity representations as inputs

	Deep models	CI	MSE	$r^2_m$
KIBA	CNNs	0.863 (0.002)	0.194	0.673 (0.009)
	SANs	0.875 (0.003)	0.179	0.691 (0.019)
	Multi-scaled SANs	**0.890 (0.002)**	**0.155**	**0.742 (0.010)**
Davis	CNNs	0.878 (0.004)	0.261	0.630 (0.017)
	SANs	0.888 (0.004)	**0.232**	**0.689 (0.007)**
	Multi-scaled SANs	**0.890 (0.005)**	0.233	0.681(0.014)

Bold values indicate the best results on the datasets

### Experiments 4: Comparison to existing approaches

In the end, we compare our multi-granularity multi-scaled SANs model to traditional methods, like KronRLS [16], SimBoost [17], and other recent deep sequence representation methods, like DeepDTA [6], MT-DTI [23], GANsDTA [20], CrossAttentionDTI [24]. Table 6 lists the results of these models on drug–target binding affinity prediction task.

**Table 6:** Results on KIBA and Davis of our proposed multi-granularity multi-scaled SANs model, transitional methods and existing deep sequence representation methods

	Method	Drug	Protein	Interaction	CI	MSE	$r^2_m$
KIBA	KronRLS [16]	Pubchem Sim	S-W	–	0.782 (0.001)	0.411	0.342 (0.001)
	SimBoost [17]	Pubchem Sim	S-W	–	0.836 (0.001)	0.222	0.629 (0.007)
	DeepDTA [6]	CNNs	CNNs	Concatenation	0.863 (0.002)	0.194	0.673 (0.009)
	MT-DTI [23]	SANs	SANs	Concatenation	0.882 (0.002)	**0.152**	0.738 (0.006)
	GANsDTA [20]	GANs	GANs	Concatenation	0.866 (−)	0.224	0.675 (−)
	CrossAttentionDTI [24]	Cross SANs	Cross SANs	Concatenation	0.874 (0.001)	0.175	–
	Ours	MSSAN	MSSAN	Concatenation	**0.890 (0.002)**	0.155	** 0.742(0.010)**
Davis	KronRLS [16]	Pubchem Sim	S-W	–	0.871 (0.001)	0.379	0.407 (0.005)
	SimBoost [17]	Pubchem Sim	S-W	–	0.872 (0.001)	0.282	0.644 (0.006)
	DeepDTA [6]	CNNs	CNNs	Concatenation	0.878 (0.004)	0.261	0.630 (0.017)
	MT-DTI [23]	SANs	SANs	Concatenation	0.887 (0.003)	0.245	0.665 (0.014)
	GANsDTA [20]	GANs	GANs	Concatenation	0.881 (−)	0.276	0.653 (−)
	CrossAttentionDTI [24]	Cross SANs	Cross SANs	Concatenation	0.876 (0.006)	0.244	–
	Ours	MSSAN	MSSAN	Concatenation	** 0.890 (0.005)**	**0.233**	** 0.681 (0.014)**

Bold values indicate the best results on the datasets

*Discussion:* As seen, these sequence-based deep models improve prediction quality than transitional methods, reconfirming the effectiveness of modeling sequence information. Besides, our proposed model improves CI to 0.890 on both KIBA and Davis dataset, and improve $r^2m$ to 0.742 and 0.681 on KIBA and Davis dataset, respectively. Thus, our model outperforms the recent sequence-based works, indicating the superiority of the proposed approaches.

## Discussion

DTI prediction is to identify the interactions between drugs and targets, which is a substantial task in the drug discovery field. Many studies proposed computation methods to reduce dependence on time, cost and traditional biological experiments. Based on these related works, we proposed a deep model for DTI prediction based on the multi-granularity encoding and the multi-scaled SAN model in this work. The main contribution of this paper can be summarized as follows.

- In order to encode fundamental chemical groups, a multi-granularity encoding method is introduced to label the molecular inputs of drugs and targets as the corresponding multi-granularity representations (Section Method).
- In order to model the multiple kinds of chemical correlations, a multi-scaled SAN model is proposed to learn the local patterns in drugs and targets by the dynamic weights (Section Method).
- Our proposed method achieves higher results on KIBA and DAVIS datasets, compared to traditional methods and recent deep sequence representation methods (Section Experiments).

Via in-depth analyses, our work may contribute to subsequent researches on this topic: (1) the multiple encoding methods of SMILES sequence and protein sequence in DTI prediction as well as other bioinformatics tasks, (2) the learning method for local patterns in sequence, and (3) the representation learning of drug and target sequences.

## Conclusion

In this paper, we investigate and propose effective approaches to improve drug–target binding affinity prediction from both encoding method and model architecture perspectives. As for the encoding method, we employ the BPE algorithm and segmentation dataset to train a multi-granularity encoding method for drug SMILES and protein sequences. It contributes to encode atoms with multiple characters and chemical functional groups. Secondly, we build a multi-scaled SAN model for their multi-granularity representations by assigning various window size to heads in original SANs. Experimental results demonstrate that the proposed approach not only is of benefit to predict DTI but also makes DTIs prediction surpass baselines on various metrics.

Our proposed method achieves the improvements by benefiting from the encoding method for chemical groups and the local patterns modeled by the representation learning model. In the encoding process, we collected a large of unlabeled data of drugs and targets to train the encoding method. Meanwhile, we found the lack of labeled data limits the improvements of deep models to predict new DTIs. Thus, our future work may focus on the utilization of these unlabeled data, like the unsupervised learning method for DTI learning.

## Data Availability

The segmentation datasets are freely available at https://pubchem.ncbi.nlm.nih.gov/ and https://ftp.expasy.org/databases/uniprot/current_release/uniparc/. The training and testing datasets for this paper are freely available at [6].

## Abbreviations

DTI: Drug–target interaction
SMILES: Simplified molecular input line entry system
OPSIN: open parser for systematic IUPAC nomenclature
GAN: Generative adversarial network
CNN: Convolutional neural network
SAN: Self-attention network
BPE: Byte-pair-encoding
NLP: Natural language processing
KronRLS: Kronecker regularized least squares
RWR: Random walk with restart
LSTM: Long-short-term memory
NCBI: National Center for Biotechnology Information
MSE: Mean squared error
CI: Concordance Index

## Footnotes

1 https://pubchem.ncbi.nlm.nih.gov/.

2 https://ftp.expasy.org/databases/uniprot/current_release/uniparc/
